# Wavefunction Engineering of Type-I/Type-II Excitons of CdSe/CdS Core-Shell Quantum Dots

**DOI:** 10.1038/s41598-018-37676-3

**Published:** 2019-01-09

**Authors:** Yashaswi Nandan, Mohan Singh Mehata

**Affiliations:** Laser-Spectroscopy Laboratory, Department of Applied Physics, Delhi Technological University, Bawana Road, Delhi, 110042 India

## Abstract

Nanostructured semiconductors have the unique shape/size-dependent band gap tunability, which has various applications. The quantum confinement effect allows controlling the spatial distribution of the charge carriers in the core-shell quantum dots (QDs). Upon increasing shell thickness (e.g., from 0.25–3.25 nm) of core-shell QDs, the radial distribution function (RDF) of hole shifts towards the shell suggesting the confinement region switched from Type-I to Type-II excitons. As a result, there is a jump in the transition energy towards the higher side (blue shift). However, an intermediate state appeared as pseudo Type II excitons, in which holes are co-localized in the shell as well core whereas electrons are confined in core only, resulting in a dual absorption band (excitation energy), carried out by the analysis of the overlap percentage using the Hartree-Fock method. The findings are a close approximation to the experimental evidences. Thus, the understanding of the motion of *e-h* in core-shell QDs is essential for photovoltaic, LEDs, *etc*.

## Introduction

Quantum dots primarily exhibits the confinement effect that leads to the spatial enclosure of the electronic charge carriers within the nanocrystals (NCs)^1^. The effect is present when the dimensions of the semiconductor are roughly below the Bohr’s exciton radius of that specific enclosure. This quantizes the conduction band (CB) and the valence band (VB) energy levels, nearly continuous for bulk semiconductors. The spacing between the valence band and conduction band can be varied by changing the size or shape of the nanocrystals which gives more monochromatic emission and near unity quantum yield by increasing shell thickness. At strong confinement regime (size below the Bohr’s exciton radius) allows for controlling the spatial distribution of carrier wave functions across various domains of a hetero-nanostructure QDs which shows size-dependent absorption and photoluminescence (PL) spectra^2,3^.Within a study carried out, the calculated exciton’s binding energy approximates to the reported Bohr’s exciton radius of CdS single crystal (2.5 nm)^4^ at the shell thickness of 2.4 nm, as obtained in our model.

The spatial separation of the electron and hole wave functions within such semiconductor heterostructure^5,6^ results in a prolonged charge transfer (CT) states which favours desirable characteristics for applications, such as light emitters, lasers, photo-catalysts and photovoltaic devices^7–12^. The charge separation state can be achieved either by direct excitation of the CT state or by photoexcitation of an exciton followed by hole transfer through the core-shell boundary. Investigating the formation and relaxation of the CT state provides information about the excitation and de-excitation processes^13^. Recently, the hetero-structuring idea has been implemented by various groups for actuating NCs capability of emitting multi-colour light because of radiative recombination of excitons confined into core/shell domains. These are DiB’s (Dot in bulk) semiconductors, which is due to the large size of the shell as compared to the core exhibits dual emission. The primary reason for dual colour emission is believed to be the size of the shell, the less non-radiative transfer of the shell’s hole towards the core, enhancing the probability of radiative recombination of the shell’s exciton into dual emission^14^. Also, the geometric separation of excitons in the nanostructures results in inhibited relaxation processes (exciton cooling and multi-exciton recombination), which gives dual-emission^15^.

Materials which has small lattice mismatch, the strain-induced localization of carriers are less efficient, as the energy levels in the CB and VB shifts insignificantly^16^. This is the case with CdSe/CdS NCs, which are structures having specific properties such as mono-dispersity, narrow emission bandwidth, and high quantum yield^17,18^. In shape altered QDs (nano-rods), it has been reported that the smaller CB and VB offset of CdSe favour localization of both electron and hole inside the core (Type-I) exciton. However, as the core size decreases, the kinetic energy of electron permits to penetrate CB offset barrier, confining over shell (Type-II) exciton^13,19–21^. This effect is predominant on the graded interface which has a step-like confinement potential at the interface. However, this separation between electron and hole is usually restricted to small-sized core quantum mechanical systems and is limited to Coulomb interaction^1,13,22^. A previous study on the differential localization of the electron and hole in the core-shell QDs is limited in the insights of the overlap integral of charge carriers for the determination of the different types of excitons and its effects on the size of the QDs. For a better understanding of the dual excitations, bulk shell structures are also needed to be studied, as it follows the same trend as predicted by our proposed model on the limitations of the dual excitations for the shell sizes^23^. In another report^24^ pseudopotential method is used to study the effect of the environment on the energy values of the electron. The environment induces excess charge carrier to imitate dielectric effect, which has been simplified by using Gauss’s law on the Hamiltonian equation to extract eigenvalues accordingly.

Controlling the spatial distribution of the charge carrier in nanorods (NRs) by altering the environment and applying an electric field, keeping the dielectric of the rod unaltered is also reported^25^. Low insulating strengths of the environment results in increased *e-h* interactions thereby increasing binding energy along with the transition probability of Type-I NRs. For the Type-II NRs, the spatial distribution of the electron completely shifts towards shell, hence transitions from pseudo-Type-II to Type-II exciton.

A novel method has been devised^26^ to compare tuning of the localization of the carrier by reduction using different photochemical *hole* quenchers (ethanol *vs*. borohydrides) of ZnO QDs. This facilitates the accumulation of *electron* in the conduction band at a rate faster than the Auger recombination without the change in charge carrier at equilibrium. By photochemical reduction using *hole* quenchers, it induces delayed response of the *e-h* recombination thereby increasing the quantum yield efficiency and the absorptivity of the QDs. A similar study^27^ has been carried out for further investigation to differentiate the regimes of electronic doping, which produces excess charge carriers and the electrostatic redox effect with equilibrium excess charge carriers. Reduced QDs produced delocalized electrons, and these electrons are produced primarily due to thermal excitations as the Fermi level is very close to the conduction band. The other regime has a Fermi level above CB, which results in a significant change of the charge carriers.

Despite previous works^15,28^ on the switch of the localization of the electron and hole in core-shell CdSe/CdS structure, these studies have ignored the explanation of the mechanism in the intermediate of e-h pair transition from Type-I exciton to Type-II exciton, specifically pseudo Type-II exciton.

Here, we have modelled 1-D core-shell (CdSe/CdS) quantum dots of radius 2–5 nm, with Hartree-Fock mean field approximation theorem solved using FDM (Finite Difference Method). The shortcoming of the Hartree-Fock Effective Mass Approximation (EMA) model in the Poisson equation was taken care of by calculating the specific e*-h* coulomb interactions without averaging it out across the domain. The mean field approximation method is limited to small QD systems. Therefore we adopted a method for calculation of Coulomb interactions known as Exact Field Approximation (EFA), which is not exploited sofar^23,28,29^. Further, the calculation of the excitation energy by the analysis of the PDF (Probability Density Function) of the electron and hole is used to discriminate the localization of the charge carriers in the core-shell domain for the size dependence variation. We studied the Schrodinger equation for the exciton, which is bounded by an electron and a hole and examined the effect of the shell thickness variation on the radial distribution functions (RDF) of both within the strong confinement regime in which the perturbation is calculated as the *e-h* Coulomb interactions. Our study focuses on the coverage of *e-h* couple from core to shell CdSe/CdS NCs that produces transitions from a Type-I to a quasi-Type-II regime and continues into the onset of the Type-II regime depending on the increase in the size of QDs.

## The Model and Theory

**Figure 1:**
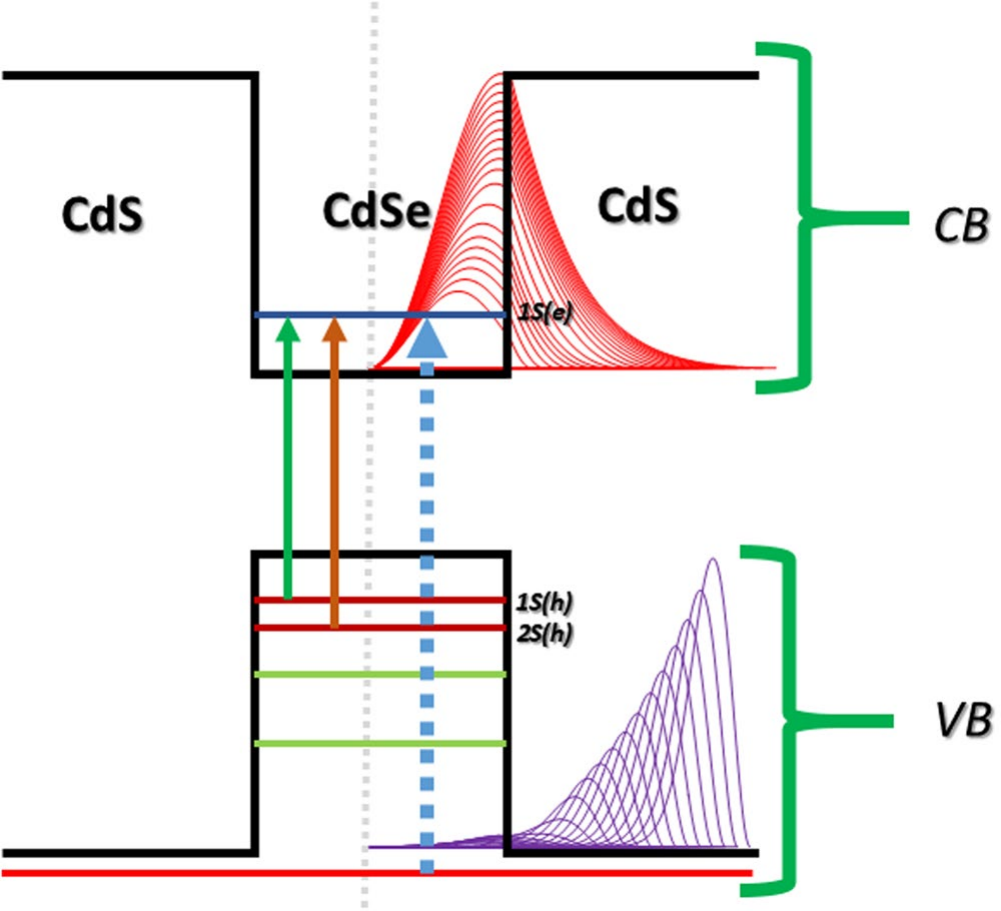
Band structure of core-shell CdSe/CdS QDs with the dual absorption (excitation) mechanism. The vertical solid line shows transitions between the core HOMO to LUMO and the dotted line between shell HOMO to core LUMO. The CB denotes the conduction band and VB denotes the valence band. Inset: PDF of electron (red) and hole (purple) are added for comparison w.r.t band structure. Demarcation dotted line in grey to point the core’s centre.

The core material, taken as CdSe with the radius ***R***_**1**_, which is passivated with a CdS shell of radius ***R***_**2**_, has a broader band gap than CdSe. The shell thickness is $\,{T}_{s}={R}_{2}-{R}_{1}$. The conduction and valence band profile of this structure is shown in the Fig. 1. The EMA (effective mass approximation) calculations (Supporting information (SI) of Method and Calculations) and BenDaniel-Duke boundary conditions were applied to solve the partial differential equations, we have assumed a 1-D spherical DLQD (Dual-layer quantum dots) heterostructure.

Schrodinger equation of an exciton is given as follows^30^:

1 $$\begin{array}{c}[-\frac{{\hslash }^{2}}{2}{\nabla }_{e}(\frac{1}{{m}_{e}^{\ast }(r)}){\nabla }_{e}-\frac{{\hslash }^{2}}{2}{\nabla }_{h}(\frac{1}{{m}_{h}^{\ast }(r)}){\nabla }_{h}-\frac{{e}^{2}}{\kappa |{r}_{e}-{r}_{h}|}+{V}_{h}(r)+{V}_{e}(r)]{\psi }_{nlm}^{exc}({r}_{e},\,{r}_{h})\\ \,\,=\,{\varepsilon }_{nlm}{\psi }_{nlm}^{exc}({r}_{e},\,{r}_{h})\end{array}$$

Here, the first and second terms are the carrier charge kinetic energies of the electron and hole, respectively. The subsequent term is the attractive Coulombic interaction energy between the electron and hole, ħ is the reduced Planck constant,$\,{{\boldsymbol{m}}}_{{\boldsymbol{e}}}^{\ast }({\boldsymbol{r}})$ and ${{\boldsymbol{m}}}_{{\boldsymbol{h}}}^{\ast }({\boldsymbol{r}})$ are the position dependent electron and hole effective masses, respectively. The effective masses of the electron and hole of the CdSe and CdS have calculated the density of state effective mass (see SI of Table S1). ***e*** is the unit coulomb charge***, κ*** is the dielectric charge of the medium, and ***V***_***e***_***(r***_***e***_***)*** and ***V***_***h***_***(r***_***h***_***)*** are the confinement potentials of the electron and hole, respectively (SI  of Table S1). The ${{\boldsymbol{\psi }}}_{{\boldsymbol{nlm}}}^{{\boldsymbol{exc}}}({r}_{e},\,{r}_{h})$ are the excitonic wave functions and the ε_nlm_ are the exciton energies. The exact solution of this PDE (partial differential equation) require huge computing resources, as the number of interacting matrix elements is approximately in the order of 10^52^ × 10^52^. This requires large storage setup, faster processing for large matrix calculation and diagonalization assuming terabytes of data flow. Therefore, some approximations have to be made^31^. In the said structure, the electron moves in a mean potential induced by the hole and similarly, the hole moves in a mean potential induced by the electron^32^. In HF (Hartree-Fock) calculations, the carriers Schrodinger equations can be simplified as^30^:

2 $$[-\frac{{\hslash }^{2}}{2}{\nabla }_{e}(\frac{1}{{m}_{e}^{\ast }(r)}){\nabla }_{e}-{q}_{e}{\varphi }_{h}+{V}_{e}(r)]{R}_{e}(r)={\varepsilon }_{e}{R}_{e}$$

3 $$[-\frac{{\hslash }^{2}}{2}{\nabla }_{h}(\frac{1}{{m}_{h}^{\ast }(r)}){\nabla }_{h}-{q}_{h}{\varphi }_{e}+{V}_{h}(r)]{R}_{h}(r)={\varepsilon }_{h}{R}_{h}$$

In these equations, *q*_*e*_ and *q*_*h*_ are the charges, and R_e_(r) and R_h_(r) represents the radial wavefunctions of the electron and hole, respectively. φ_e_(φ_h_) is the Coulombic potential generated by the electron (hole). The two PDEs are used to solve the radial part; the angular part has been omitted as we constrain ourselves to the PDFs of the e-h pair (exciton). As evident from the Schrodinger equations, we ignored XC (Exchange-Correlations) effects as they hardly contribute to the electronic energies (see SI of methods and calculations).

The confinement potential for the electron between R_1_ and R_2_ (eq. 5) is calculated by the difference of the electron affinity of the core and the shell. Similarly, for the hole (eq. 6), it is calculated by reducing confinement potential of electrons with the difference of band gap between core and shell^33^. Beyond R_2_, the QD is assumed to be in an infinite potential wall, this amounts to a node for the electron and hole wavefunctions at the periphery of the spherical QD. The confinement potential profile of QDs is represented as:

4 $${V}_{e,h}=\{\begin{array}{ll}0, & \,r < {R}_{1}\\ {V}_{e,h}, & {R}_{2}\le r\le {R}_{\begin{array}{c}1\end{array}}\\ \infty , & r > {R}_{2}\end{array}\}$$

5 $${V}_{e}={\chi }_{CdSe}-{\chi }_{CdS}$$

6 $${V}_{h}={E}_{bg(CdS)}-{E}_{bg(CdSe)}-{V}_{e}$$

Here, we have assumed the interface between core and shell to be step-like or non-alloyed. Therefore, the confinement potential is assumed constant for the core and shell domains. The surface edge effects and mismatch effects were ignored due to small lattice mismatch (See SI of methods and calculations).

For the interface between the domains, the BenDaniel–Duke boundary conditions^34,35^ is applied as:

7 $$|{R}_{e,h}(r),{|}_{r={R}_{1}}=|{R}_{e,h}(r){|}_{r={R}_{2}}$$

8 $${|\frac{1}{{m}_{e,h}^{\ast (CdSe)}}\frac{d{R}_{e,h}(r)}{dr}|}_{r={R}_{1}}={|\frac{1}{{m}_{e,h}^{\ast (CdS)}}\frac{d{R}_{e,h}(r)}{dr}|}_{r={R}_{2}}$$

Equation (7) represents the continuity of the RDF across the interface in the zeroth order differential. The BenDaniel-Duke boundary conditions cause the continuity of the probability current densities at the interface (eq. 8). Here, the effective mass of the charge carriers at core and shell (See SI of Table S1) are different across the interface^36^, therefore, the boundary conditions compensates the effective mass mismatch at the interface unlike in the case of classical boundary equations which is introduced for the continuity of the radial wavefunctions of the first order differential.

The electrostatic potentials induced by the electron and hole can be solved by the Poisson equations, as given below^30^:

9 $$\overrightarrow{{\nabla }}\kappa (r)\overrightarrow{{\nabla }}{\varphi }_{e}={q}_{e}{\rho }_{e}$$

10 $$\overrightarrow{{\nabla }}\kappa (r)\overrightarrow{{\nabla }}{\varphi }_{h}=-\,{q}_{h}{\rho }_{h}$$

here, ***ρ***_***e***_***(r)*** and ***ρ***_***h***_***(r)*** are the linear carrier charge densities, and ***κ(r)*** is the position dependent bulk dielectric constant of the core and shell of the QD. Equations 9 and 10 models the electronic potential images which arise due to surface polarization at the core-shell boundaries. The charge carriers (e–h) linear densities are as follows^33^:

11 $${\rho }_{e}=\frac{1}{4\pi }q|{R}_{e}^{n,l}(r){|}^{2}$$

12 $${\rho }_{h}=\frac{1}{4\pi }q|{R}_{h}^{n,l}(r){|}^{2}$$

Here, *n* represents the principal quantum number and *l* is the azimuthal quantum number. Since we are concerned with the modelling of s-s transitions for the excitations, therefore we have assumed *n* = *1* and *l* = *0* for both the electron as well as a hole.

## Results and Discussion

**Figure 2:**
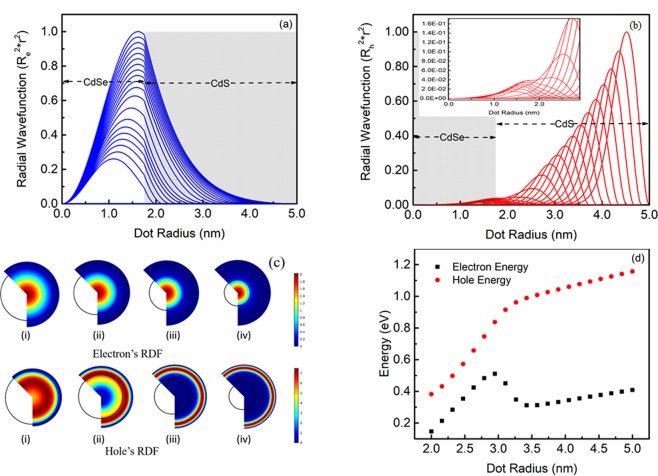
The probability density function  of the electron (**a**) and hole (**b**) consists of multiple curves, each RDF curve is plotted as a function of the QDs radius starting from 2 to 5 nm for a single exciton. The inset of (b) shows the magnified curves for the probability density function of the hole in the smaller dot. (**c**) Graphic representation of the electron and hole RDFs of 1.75 nm CdSe core along with a shell thickness of 0.25 (i) 1.25 (ii) 2.25 (iii) and 3.25 nm (iv). The electron’s radial wavefunctions are localized over the core domain consistently and the hole’s shift towards the shell. (**d**) Energies of the electron and the hole with varying shell thickness.

The radial distribution functions (RDF) calculated using the Hartree-Fock mean field approximation method represents the spatial distribution of the charge carriers in QDs in steady-state. Figure 2(a–c) shows the behaviour of the distribution of the electron and the hole RDF of CdSe/CdS QD with the increase of the shell thickness from 0.25–3.25 nm with a fixed core radius of 1.75 nm. The electron’s spatial distribution is delocalized across the whole domain due to small effective mass which amounts to high kinetic energy, further facilitated by low band offset of the conduction band (V_e_ = 0.32 eV), making delocalization of the electron into the shell easier. The peak of the curve becomes sharp and increases within the QDs as the shell thickness increases to 1.2 nm (Fig. 2(a)). The sharpness of the electron’s RDF is independent of the spatial distribution of the hole since hole and electron are co-localized in the same core region (Type-I exciton) till 1.2 nm. The localization of the electron in QDs is governed by the shell size, which acts as a confinement potential of the electron inhibiting the electron’s tunnelling probability from the core towards the shell (Fig. 2a). Beyond 1.2 nm shell thickness, electron’s energy decreases, as the full-width half-maximum (FWHM) of the electron RDF is reduced (Fig. 2(d)), demonstrated the hole (+*ve*) and electron (*−ve*) energy curves as a function of QD radius, overlapped into the same region to compare the separation of (e-h) energy. Here, the distribution of hole plays an important role. It is shifting towards the shell from the core; this exerts a Coulumbic force on the electron to attract it towards the shell. Also, there is repulsion between the core localized excitons and the shell localized excitons, as the hole are virtually electronically decoupled from each other and separated by an energy gap below a quasi-continuum of shell’s energy levels^5,37^ and dynamic coulomb blockade process is responsible for the hindering the relaxation of shell localized holes to the core states for sharp confinement potentials^14^. As a result, the electron’s spatial distribution is broadened, which causes a reduction in the sharpness of the peak of the spatial distribution of the electron, and the similar trend is followed till 1.8 nm shell thickness.

Upon further increasing shell thickness (above 1.8 nm), the sharpness of the RDF regains because the hole RDF has completely shifted from the core towards shell. Therefore, the Coulomb interaction between the electron and the hole is static due to confinement of hole in shell completely. Due to this, there is a negligible Coulombic effect on the electron, hence it follows general localization of the electron in the entire domain.

Figure 2(b) shows the hole’s probability density function, which is shifting from core to the shell, causing the overlap probability (exciton) to shift towards the shell w.r.t shell coverage. Hole’s energy monotonically increases with the increase in shell passivation. Since the hole is confined towards the shell making the radial distribution function more sharper thereby enhance the energy of the hole. In the intermediate state, the hole RDF is localized in the core as well as in shell after the shift due to high effective mass amounting to lower kinetic energy, this intermediate region is known as the pseudo-Type-II exciton. This is facilitated further by low valence band confinement potential (V_h_ = 0.43 eV), which makes the hole easier to penetrate towards shell. This shift is instrumental to the reasons for various effects including the excitation’s energy variation and specifically in understanding the transition from Type-I to Type-II excitons.

**Figure 3:**
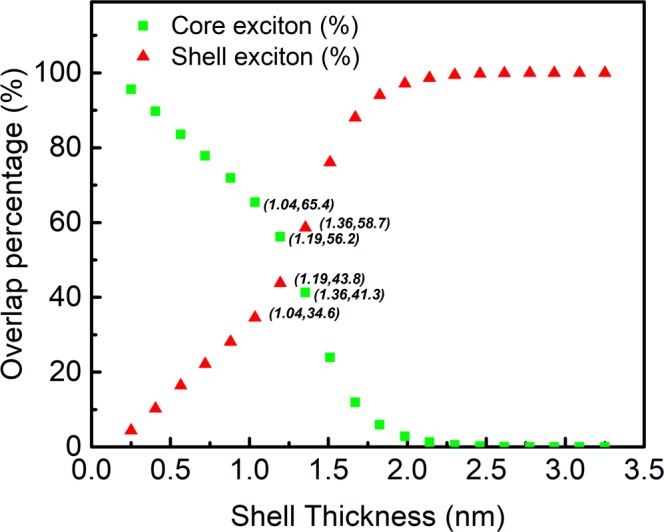
The probability distribution of single exciton (electron-hole) as a function of shell thickness upto 3.25 nm (5.0 nm QD radius) with respect to the percentage area covered by both the core and the shell excitons of the QDs. The number in parenthesis is the shell thickness and overlap percentage.

Relative overlap percentage is a unique way to know the radial density distribution of the exciton over the core-shell to establish the confinement region for excitation energies calculations. The core exciton is decreasing as the thickness of the shell is increased (Fig. 3), since our concern is with the boundary limit 70.7% of core-shell exciton concentration in core-shell QDs to discriminate the different exciton types. Below 0.88 nm shell thickness, the concentration of the core excitons w.r.t. the total concentration of the excitons is above 70.7% (Type-I exciton). This suggests a shift in the overlap probability of the excitons that can be engineered by applying external electric fields^38^.

There are data points between shell thicknesses 1.04 to 1.36 nm (Fig. 3) for which the overlap percentage of the core-shell below 70.7%, which may exhibit dual absorption, two transition energies for each shell thickness suggesting dual absorption from the pseudo-type-II exciton. After this intermediate regime (shell thickness: 1.04–1.36 nm), the confinement region of exciton shifts towards the shell, known as Type-II exciton (Fig. 3). The Type-II exciton produces an absorption which corresponds to the energy difference between core conduction band and the shell valence band (shell to core transitions, Fig. 1).

**Figure 4:**
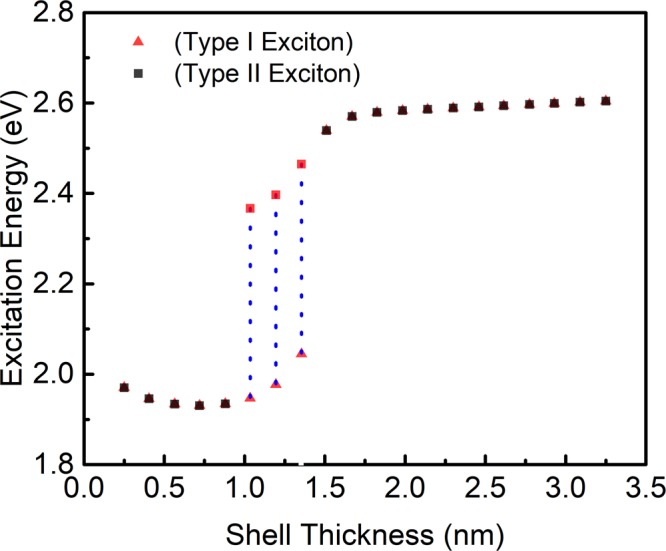
The excitation energies of the excitons with varying shell thickness. The blue vertical dotted lines are representing the dual absorption region.

In the calculation of the excitation energy for dual absorption (See SI of methods and calculations), we took both the bulk bandgaps of the core CdSe and the difference between the conduction band of core and the valence band of the shell (pseudo-Type-II exciton). Similar effects were observed experimentally for the QDs as reported in the literature^13,39,40^. The excitation energy is decreasing with increasing shell width up to 0.72 nm, the decrease is about 3 meV, as shown in Fig. 4. This arises due to the increase in the width of the shell (CdS), also the excitation energy is inversely related to the square of the confinement region’s dimension, which agrees to the confinement effect. For 1.04–1.36 nm shell thickness, the curve shows dual excitations with a substantial difference in excitation energy. The overlap percentage of the core or the shell confined exciton w.r.t. each other is below 70.7% in this region. Above 1.67 nm shell thickness, the rate of increase of excitation energy is stagnant after a jump in the excitation energy, due to switching into Type-II exciton having electron delocalized in the core and the hole completely localized in the shell (Fig. 2c). There is no significant increase in the transition energy change w.r.t. shell thickness as the *e-h* pairs are fully confined. The dual emission exhibited by ultra-thick CdSe/CdS core-shell QDs can also be related to this model for further investigations. This effect is primarily due to the thick shell QDs and the nature of the interface between the core and the shell; the step-like confinement potential is more bound to show this effect than the smooth confinement potential^14^.

**Figure 5:**
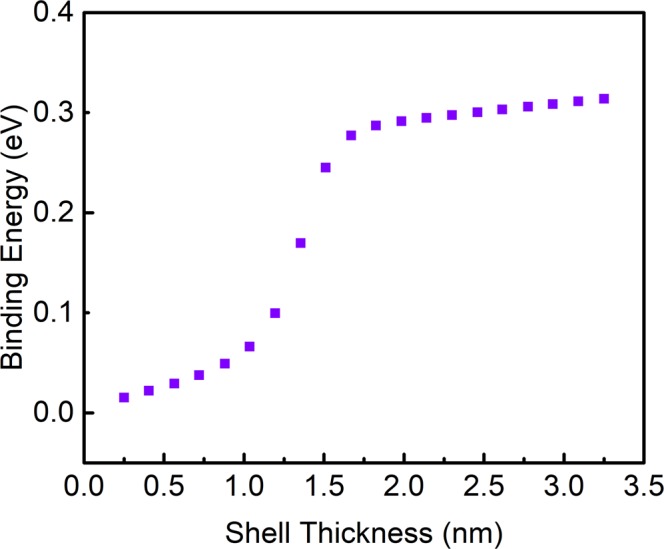
The binding energy of exciton as a function of shell (CdS) thickness.

The binding energy of excitons was estimated and are shown in Fig. 5. It has been observed that the values are positive for each shell thicknesses suggesting attractive interactions between the hole and electron. Variation of these interactions are the physical reasons for variation of the binding energy with the shell thickness. The rise in binding energy can be understood predominantly by the increase in the electron and hole probability densities. Therefore, the interactions between them increase. Above 1.36 nm shell thickness, the hole’s probability density starts to shift towards the shell from core, since the electron remains confined in the core, therefore, with the increase in probability density of both, the hole-electron separation is accelerated due to the shifting of hole and after the full confinement of the hole, the hole shifts along with the shell thickness increase, which further takes the hole away from the core or here, the core confined electrons. This causes the reduction of the electron and hole Coulombic interaction showing a steep increase in the binding energy^40^ (positive slope) till 1.67 nm. After 1.67 nm shell thickness, the separation between them does not increase at a fast rate signifying that the slope of the binding energy becomes less sharp as the shell thickness increases.

**Figure 6:**
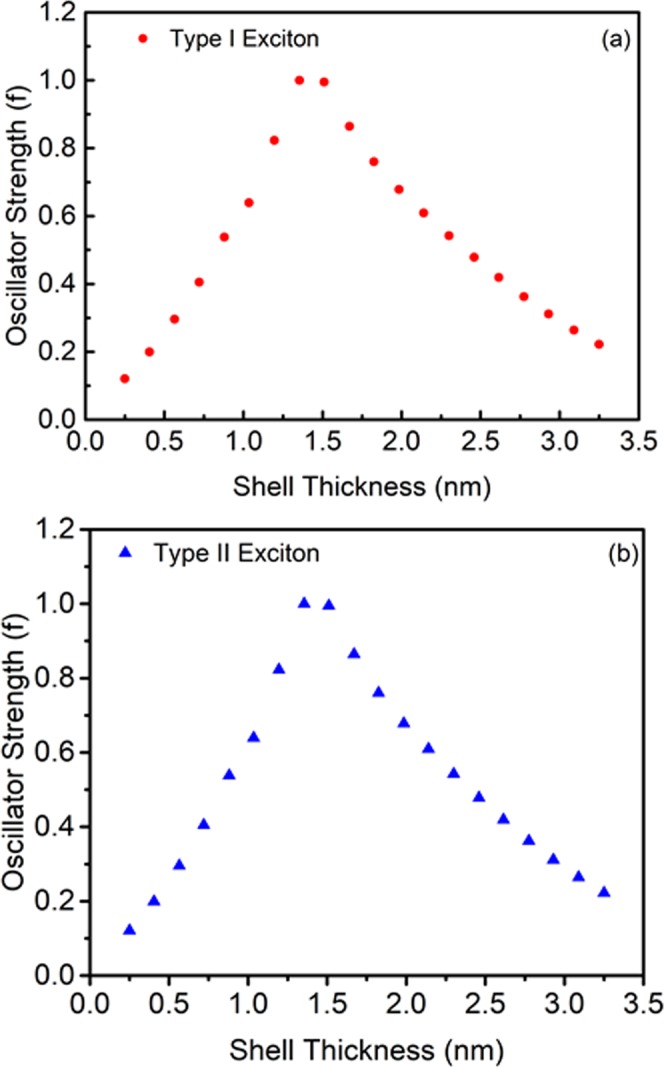
The oscillator strength of Type I (**a**) and Type II (**b**) excitons as a function of the shell thickness in the gas phase.

The absorption intensity was determined theoretically in terms of the oscillator strength. Figure 6(a,b) gives the normalized oscillator strength of QDs for different shell thickness in the gas phase for both the (a) Type-I (eh|) confinement and (b) Type-II (e|h) confinement. The oscillator strength for second excitation peaks was also estimated for the transitions from 2 S(h) of VB to 1S(e) of CB. The electrons excited from the second sub-band of the valence band (HOMO-1) to the conduction band of the LUMO (Fig. 1). The second excitation state has relatively less oscillator strength due to the negative curve of the hole radial wave function for the second sub-band level of the valence band having a single node. The overlap integral of the second excitation is lower than the first excitation peak due to destructive interference (SI of Fig. S1). Thus, the oscillator strength follows the same trend^11^. Further, the simulated relative oscillator strength was compared^12,31^ with the normalized experimental absorption spectrum obtained for CdSe/CdS QDs prepared using chemical route at high temperature. The core size of synthesized QDs was typically 1.75 ± 0.25 nm and the shell thickness was 0.25 ± 0.25 nm, which produces the first absorption (excitation) peak at 547 nm^12^ and 590 nm^31^ and the corresponding simulated value was obtained at 542 nm. The second excitation peak was observed experimentally at 438 nm and theoretically at 481 nm. The difference in the excitation energy might be due to the non-uniformity (shape) of the core-shell structure prepared experimentally, 1-D modelling and also due to the different environments, i.e., the liquid phase for the experimental and gas phase for theoretical.

**Figure 7:**
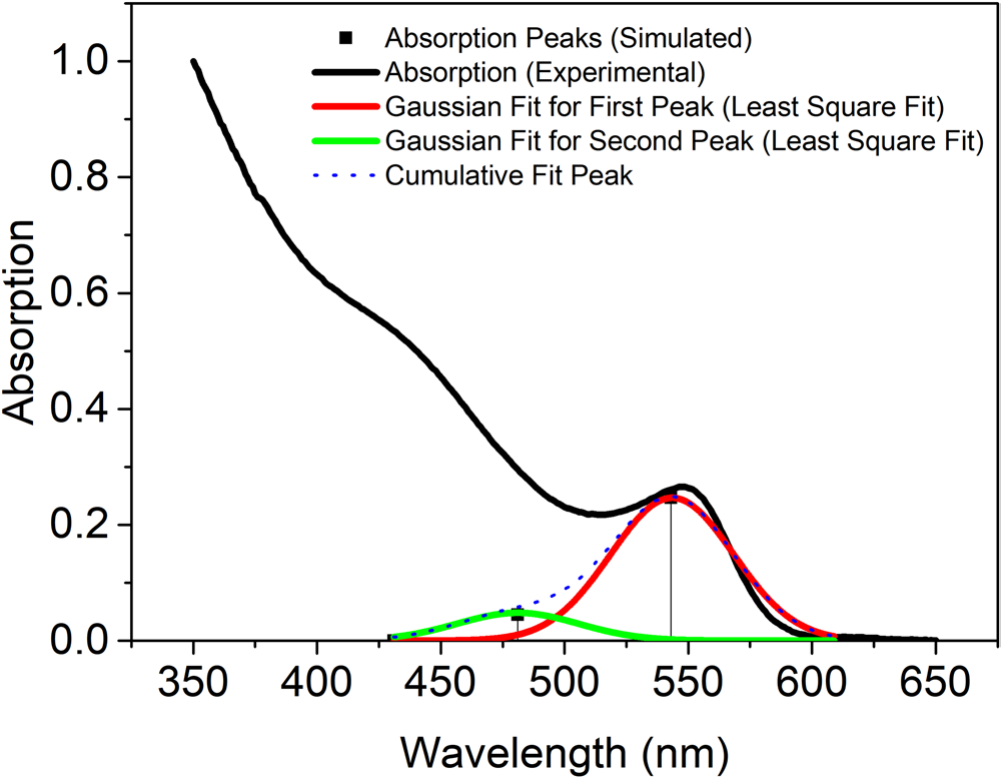
The fitted Gaussian curves on simulated peaks overlapped with the experimental absorption spectrum of CdSe/CdS QDs.

According to our model, the shell thickness for the particular excitation energy matches with 0.25 nm, which is well within the allowed error estimation of about 0.2/0.15 eV^12,31^. The multiple peak fit is employed to simulate Gaussian curves to represent the calculated excitation peaks and superposed on the actual absorption curve along with the summated Gaussian curve for the first and second excitation bands (Fig. 7). It is important to mention that the modelled QDs follow the similar trend, i.e., the excitation energies decrease with increasing the shell thickness up to 3–4 monolayers^37^ (e.g., 1 ML = 0.25 nm).

**Figure 8:**
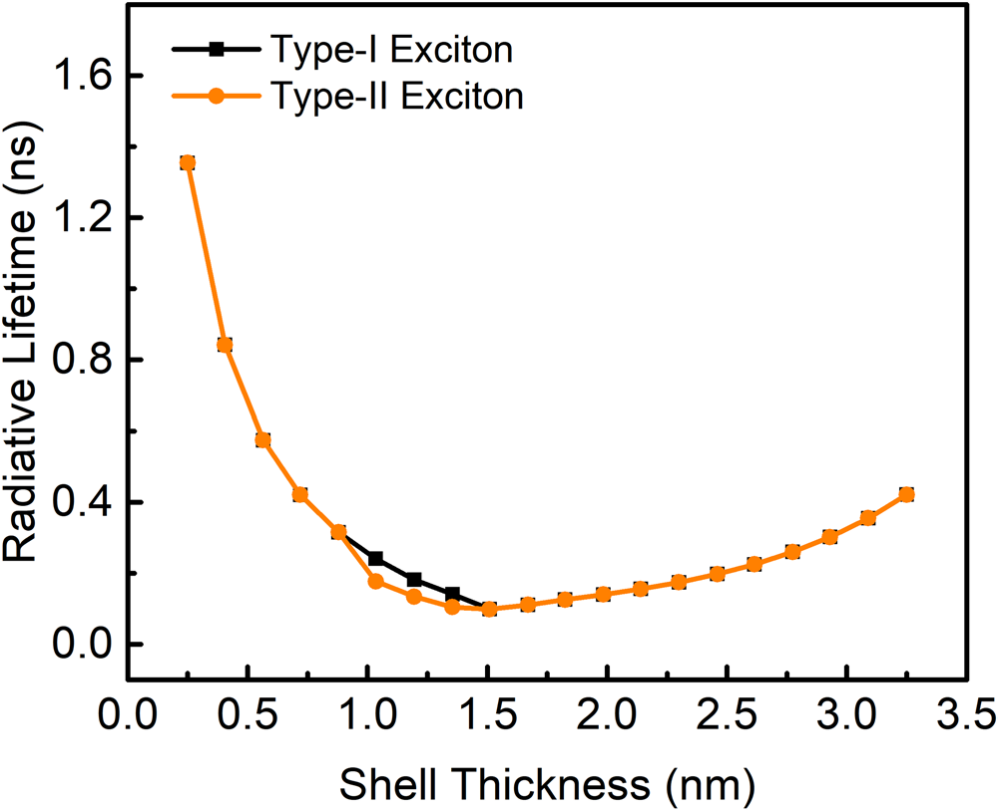
An estimated natural lifetime of excitons (Type I and II) as a function of shell thickness.

Figure 8 shows the variation of radiative (natural) lifetime time as a function of shell thickness estimated using the equation provided in supplemental information (Methods and Calculations). As the shell thickness increases from 0.2–1.4 nm, the lifetime decreases and become nearly constant afterwards with slight increase. This trend is quite convincing as per the reported^12,41^ experimental values. There are evidences^42,43^ for this trend at very low temperatures, e.g., at about 1.5 K which arises due to negligible non-radiative rates. The inorganic shell passivation leads to decrease in lifetime^44^, as the overlap integral between electron and hole increases overall^23^, since there is increase in electron’s RDF in the core region and the increase in the spread of the hole’s RDF in the region of the Type-I QDs. Beyond Type-I QD structure, the increase in shell thickness, there is a decline in the overlap integral of the e-h pair which constitutes for the increase in the radiative lifetime^45^. Many reports^41,42,45^ have confirmed that the shell passivation increases the quantum yield of the QDs, which is related to the decrease of the radiative lifetime, as the non-radiative rates becomes less^45^. This is due to, the Auger decay mitigation, as the shell blocks electrons transfer into the dot^46^. Typically, for 0.25 nm shell thickness, the calculated lifetime is 1.35 ns, whereas the radiative lifetime of the experimentally prepared QDs is 0.39 ns^41^/2.18 ns^12^. The difference of the lifetimes is compensated primarily due to the 1-D description used in our modelling, ignoring of non-radiative recombinations, the synthesized QDs may not be perfectly spherical, may contain defect states and the environment (dielectric) effects. Thus, the radiative and non-radiative transitions are quite relevant parameters for the device applications, e.g., solar cells, LEDs, photo-detectors, etc.

## Conclusions

**Figure 9:**
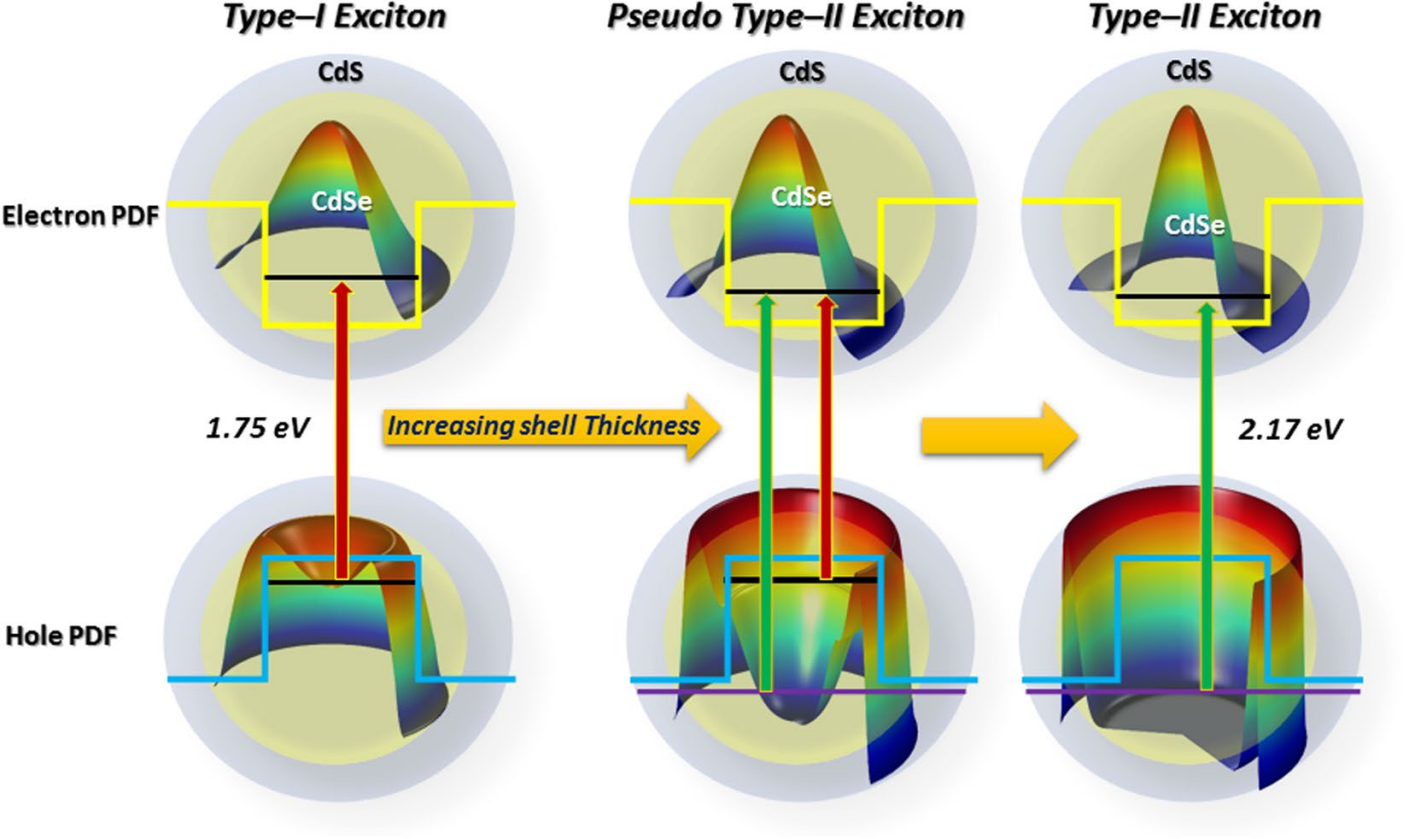
Graphical representation of type I, pseudo type-II and type-II excitons

Theoretical modelling was employed to obtain the spatial distribution of charge carriers in the core-shell QDs. A charge carries separation between the core confined, and shell confined excitons were obtained above the four monolayers (with a fixed core size QDs). However, beyond six monolayers, the binding and excitation energies are not size-sensitive. In addition to the type-I and type-II excitons, pseudo-type-II excitons (as illustrated in Fig. 9) were obtained within specific shell thickness. The pseudo-type-II excitons lead to the dual absorption bands, which may also give dual emission in the step-like confinement potential^13^. Though the mechanism suggested is debatable and requires much attention on this front for further explanation. We would like that this study will contribute a lot to understanding the basis of electronic and optical properties of the excitons in multi-layered heterostructure QDs and will provide information for material fabrication.

## Supplementary information

Supplementary Information

## References

[1] Rainò G (2011). Probing the wave function delocalization in CdSe/CdS dot-in-rod nanocrystals by time-and temperature-resolved spectroscopy. ACS Nano.

[2] Laheld UEH, Einevoll GT (1997). Excitons in CdSe quantum dots. Physical Review B.

[3] Tsuchiya T (2000). Biexcitons and charged excitons in quantum dots: A quantum Monte Carlo study. Physica E: Low-Dimensional Systems and Nanostructures.

[4] Pokutnii SI (2010). Exciton binding energy in semiconductor quantum dots. Semiconductors.

[5] Huynh WU (2003). Charge transport in hybrid nanorod-polymer composite photovoltaic cells. Physical Review B.

[6] Acharya KP (2009). Linker-free modification of TiO2Nanorods with PbSe nanocrystals. The Journal of Physical Chemistry C.

[7] Hyun B (2011). Electron injection from colloidal PbS quantum dots into titanium dioxide nanoparticles. ACS Nano.

[8] Acharya KP (2011). Synthesis of PbS/TiO2Colloidal heterostructures for photovoltaic applications. The Journal of Physical Chemistry C.

[9] Kumar S, Jones M, Lo S, Scholes G (2007). Nanorod heterostructures showing photoinduced charge separation. Small.

[10] Aktürk A, Sahin M, Koc F, Erdinc A (2014). A detailed investigation of electronic and optical properties of the exciton, the biexciton and charged excitons in a multi-shell quantum dot nanocrystal. Journal of Physics D: Applied Physics.

[11] Smith AM, Lane LA, Nie S (2014). Mapping the spatial distribution of charge carriers in quantum-confined heterostructures. Nature Communications.

[12] Ratnesh RK, Mehata MS (2017). Investigation of biocompatible and protein sensitive highly luminescent quantum dots/nanocrystals of CdSe, CdSe/ZnS and CdSe/CdS. Spectrochimica Acta Part A: Molecular and Biomolecular Spectroscopy.

[13] Chuang C, Doane TL, Lo SS, Scholes GD, Burda C (2011). Measuring electron and hole transfer in Core/Shell nanoheterostructures. ACS Nano.

[14] Pinchetti V (2016). Effect of Core/Shell interface on carrier dynamics and optical gain properties of dual-color emitting CdSe/CdS nanocrystals. ACS Nano.

[15] Kim S, Fisher B, Eisler H, Bawendi M (2003). Type-II quantum dots: CdTe/CdSe (Core/Shell) and CdSe/ZnTe (Core/Shell) heterostructures. Journal of the American Chemical Society.

[16] Segarra C (2016). Piezoelectric control of the exciton wave function in colloidal CdSe/CdS nanocrystals. The Journal of Physical Chemistry Letters.

[17] Garcia- Santamaría F (2009). Suppressed auger recombination in “giant” nanocrystals boosts optical gain performance. Nano Letters.

[18] Garcia-Santamaría F (2011). Breakdown of volume scaling in auger recombination in CdSe/CdS heteronanocrystals: The role of the Core-Shell interface. Nano Letters.

[19] Eshet H, Grünwald M, Rabani E (2013). The electronic structure of CdSe/CdS Core/Shell seeded nanorods: Type-I or quasi-type-II?. Nano Letters.

[20] Shabaev A, Rodina AV, Efros AL (2012). Fine structure of the band-edge excitons and trions in CdSe/CdS core/shell nanocrystals. Physical Review B.

[21] Jaskolski W, Bryant GW (1998). Multiband theory of quantum-dot quantum wells: Dim excitons, bright excitons, and charge separation in heteronanostructures. Physical Review B.

[22] Lo SS, Mirkovic T, Chuang C, Burda C, Scholes GD (2010). Emergent properties resulting from type-II band alignment in semiconductor nanoheterostructures. Advanced Materials.

[23] Kocevski V, Rusz J, Eriksson O, Sarma DD (2015). First-principles study of the influence of different interfaces and core types on the properties of CdSe/CdS core-shell nanocrystals. Scientific Reports.

[24] Liu H (2017). A hybrid quantum-classical model of electrostatics in multiply charged quantum dots. The Journal of Physical Chemistry C.

[25] Royo M, Climente JI, Movilla JL, Planelles J (2010). Dielectric confinement of excitons in type-I and type-II semiconductor nanorods. Journal of Physics: Condensed Matter.

[26] Schimpf AM, Gunthardt CE, Rinehart JD, Mayer JM, Gamelin DR (2013). Controlling carrier densities in photochemically reduced colloidal ZnO nanocrystals: size dependence and role of the hole quencher. Journal of the American Chemical Society.

[27] Schimpf AM, Knowles KE, Carroll GM, Gamelin DR (2015). Electronic doping and redox-potential tuning in colloidal semiconductor nanocrystals. Accounts of Chemical Research.

[28] Balet LP, Ivanov SA, Piryatinski A, Achermann M, Klimov VI (2004). Inverted core/shell nanocrystals continuously tunable between type-I and type-II localization regimes. Nano Letters.

[29] Wang LW, Zunger A (1996). Pseudopotential calculations of nanoscale CdSe quantum dots. Physical Review B.

[30] Åžahin M, Nizamoglu S, Yerli O, Volkan Demir H (2012). Reordering orbitals of semiconductor multi-shell quantum dot-quantum well heteronanocrystals. Journal of Applied Physics.

[31] Li JJ (2003). Large-scale synthesis of nearly monodisperse CdSe/CdS Core/Shell nanocrystals using air-stable reagents via successive ion layer adsorption and reaction. Journal of the American Chemical Society.

[32] Christodoulou S (2014). Synthesis of highly luminescent wurtzite CdSe/CdS giant-shell nanocrystals using a fast continuous injection route. Journal of Materials Chemistry C.

[33] Åžahin M, Nizamoglu S, Kavruk AE, Demir HV (2009). Self-consistent computation of electronic and optical properties of a single exciton in a spherical quantum dot via matrix diagonalization method. Journal of Applied Physics.

[34] Luo J, Bester G, Zunger A (2009). Long- and short-range electron-hole exchange interaction in different types of quantum dots. New Journal of Physics.

[35] Peleshchak, Bachynsky (2009). Electric properties of the interface quantum dot - matrix. Condensed Matter Physics.

[36] Barsan V, Ciornei MC (2016). Semiconductor quantum wells with BenDaniel–Duke boundary conditions: approximate analytical results. European Journal of Physics.

[37] Klimov VI (2007). Single-exciton optical gain in semiconductor nanocrystals. Nature.

[38] Mehata MS (2015). Enhancement of charge transfer and quenching of photoluminescence of capped CdS quantum dots. Scientific Reports.

[39] Mukherjee A, Ghosh S (2012). Optimum excitation photon energy for CdSe-ZnS core-shell quantum dot based luminescence imaging. Journal of Physics D: Applied Physics.

[40] Sarkar P, Springborg M, Seifert G (2005). A theoretical study of the structural and electronic properties of CdSe/CdS and CdS/CdSe core/shell nanoparticles. Chemical Physics Letters.

[41] Ratnesh RK, Mehata MS (2017). Synthesis and optical properties of core-multi-shell CdSe/CdS/ZnS quantum dots: Surface modifications. Optical Materials.

[42] Brovelli S (2011). Nano-engineered electron–hole exchange interaction controls exciton dynamics in core–shell semiconductor nanocrystals. Nature Communications.

[43] Javaux C (2013). Thermal activation of non-radiative Auger recombination in charged colloidal nanocrystals. Nature Nanotechnology.

[44] Hines MA, Guyot-Sionnest P (1996). Synthesis and characterization of strongly luminescing ZnS-capped CdSe nanocrystals. The Journal of Physical Chemistry.

[45] Peng X, Schlamp MC, Kadavanich AV, Alivisatos AP (1997). Epitaxial growth of highly luminescent CdSe/CdS core/shell nanocrystals with photostability and electronic accessibility. Journal of the American Chemical Society.

[46] Bae WK (2013). Controlling the influence of Auger recombination on the performance of quantum-dot light-emitting diodes. Nature Communications.

