# Supplementary material for: Wavefunction Engineering of Type-I/Type-II Excitons of CdSe/CdS Core-Shell Quantum Dots
Source: Sci Rep. 2019 Jan 9;9:2. doi: 10.1038/s41598-018-37676-3 (PMC6327053; doi:10.1038/s41598-018-37676-3)
Supplement: Supplementary file 1 — Supplementary Information [file 41598_2018_37676_MOESM1_ESM.pdf]

Supporting Information

Wavefunction Engineering of Type-I/Type-II Excitons of CdSe/CdS Core-Shell Quantum Dots

Yashaswi Nandan, Mohan Singh Mehata*

Laser-Spectroscopy Laboratory, Department of Applied Physics, Delhi Technological University, Bawana Road, Delhi-110042, INDIA.

*Corresponding author E-mail: msmehata@gmail.com; msmehata@dtu.ac.in

Methods and Calculations:

Atomic units have been used throughout the calculations, in which Planck's constant ħ and the electronic charge are unity, and the bare carrier charge mass is in a.u. The core radius of the CdSe is fixed at 1.75 nm for all the calculations, and the shell thickness of the CdS has been varied stepwise on a parametric sweep between 0.25 nm to 3.25 nm thickness of the shell over 20 cycles. An exciton is bound to each other via the Coulomb interaction and other exchanges and correlation interactions are also present. The Schrodinger equations of the electron and hole are given in equations (2) and (3), described under the model and theory section of the manuscript. As can be inferred from the equations (2) and (3), the electron is restricted by the confinement potential, $V_e(r)$, and the attractive Coulomb potential, $-q_e\varphi_h$, originated by the hole acting on it. Correspondingly, the hole is restricted by the confinement potential, $V_h(r)$, and the attractive Coulomb potential, $-q_h\varphi_e$, exerted by the electron (as shown in Fig. S2). The XC (exchange-correlation) energy of the electron-hole couple is present, even if it is very low[1,2,3]. In CdSe semiconductor, the XC energy of the electron and hole is approximately 0.13 meV[1,4] and, therefore, it is insignificant and can be ignored in the calculations. Also, the lattice mismatch between CdSe and CdS is about 4% (CdSe lattice parameter: 4.3 Å, CdS lattice parameter: 4.136Å). This results in negligible strain and defect probability. The small lattice mismatch gives the possibility to grow thick shells with few strain-induced defects.[5] Since in our calculations we have ignored certain effects such as correlation and exchange therefore, to validate our arguments assumption the DFT calculations were performed for the CdSe Zinc-Blende structure QDs considering 1 nm size using the hybrid functional PBE1 (Perdew–Burke–Ernzerhof)[6,7] model as the most appropriate functional reported in a work[8] and optimize the geometry with STO-3G basis set. The molecular setup we designed is a hyper-cell of a-axis (8.6 Å), b-axis (8.6Å) and c-axis (14.04 Å), stoichiometry of $Cd_{16}Se_{16}$. Gaussian 09 software package[9] was used for single point energy (SPE) calculations, we obtained the direct band gap

energy in the range of 1.49 to 3.85 eV at different k-points, which is very similar to that obtained (2.7 eV) using our proposed model for pure CdSe QDs. The correlation value calculated by the DFT comes in the order of 10^{-4} eV (0.07 meV) and the exchange value is in the order of 10^{-2} eV (3.7 meV). These values can be ignored as the Coulomb interaction is the predominant factor, which contributes to the direct band gap energy. Therefore, the calculations for Coulombic interaction effects of the excitons were performed.

Further to verify the veracity of the model, regression analysis of the eigenvalue and the wavefunction of the charge carriers for ultra-thin shell and the pure core QDs were carried out. The Adj. R^2 value came nearly unity for both the electron and hole probability density function (PDF) showing nearly full recovery between the pure core and core-shell (ultra-thin shell) QDs. Both the parameters were analyzed using the multiple linear regression model.

MUMPS (multi massively parallel sparse direct solver) has been employed to solve the Schrödinger's partial differential equation provided in COMSOL Multiphysics[10] software package. The QDs are solved in a one-dimensional (1-D) array, spaced equally at 0.005 nm each, this array contains equally spaced nodes which are considered as an electron or a hole, and have taken that there is one exciton per NCs. Using this, the overlap probability is determined, and the excitation energy is consequently calculated from the energy (eigenvalues) solving Schrödinger's equation.

The Schrödinger equation is solved using Hartree-Fock self-consistent mean field approximation due to the mean potential field applied by the charge carriers on each other. Initially, the electron and hole are taken as non-interactive, as there are no Coulomb forces applied to the carriers, the Schrodinger's equation is solved for them individually in an iteration. Using Poisson's equations, the Coulomb interaction between the electron and hole is calculated from initial wave functions, which further modifies the confinement potential in the Schrödinger's equation at each cycle.[11] The self-consistent loop is controlled using COMSOL

live link with MATLAB[12] (Fig. S3). This programming is done to calculate the convergence between the initial and new wavefunctions and terminate the loop when convergence is attained. The electronic and optical properties, such as the binding energies, excitation energies, overlap integrals, overlap probabilities, oscillator strengths, the radial density of wave function, etc., are determined using the Origin analysis software package. The calculation of the overlap probability and the subsequent integration of the overlap probability with respect to the radius of the QDs is done on the Origin software.[13] Integration was carried out by package using the simple trapezoidal method. This approach is more accurate as the density of the data is high.

Electronic properties

The total energy of the single exciton is defined as[14,15]:

$$E_X^{total} = E_g + \varepsilon_e + \varepsilon_h - \varepsilon_{e-h} \tag{1}$$

Here, $\boldsymbol{E_g}$ is the band gap energy of the confinement region. The confinement region of the exciton is selected according to the relative integral of overlap probability of the exciton in the core-shell with respect to the QDs. In modeling, we have evaluated the perturbation by the Coulomb interaction, since the dimensions of the QDs semiconductor is within the Bohr's exciton radius for CdSe ($\boldsymbol{R_{Bohr}}$ = 5.6 nm).[16] The percentages of the overlap area of the core-shell regions were evaluated with respect to the QDs. The eigenvalues ($\boldsymbol{\varepsilon_e, \varepsilon_h}$) were determined at the termination of the loop, $\boldsymbol{\varepsilon_{e-h}}$ is the attractive Coulomb energy, between the electron and hole. Since the attractive Coulomb energy is added twice (one is from $\boldsymbol{\varepsilon_e}$ and the other from $\boldsymbol{\varepsilon_h}$), thus, one $\boldsymbol{\varepsilon_{e-h}}$ is subtracted from the total energy to rectify the error. The attractive Coulomb energy is determined using:[17]

$$\varepsilon_{e-h} = -\frac{(\varepsilon_e^0 - \varepsilon_e + \varepsilon_h^0 - \varepsilon_h)}{2} \tag{2}$$

In Eq. 2, ε_e^0 and ε_h^0 are the single electron and single hole energies calculated in the first cycle taken without Columbic interactions. The exciton binding energy is defined as:[18,19]

$$E_b(X) = E_g + \varepsilon_e^0 + \varepsilon_h^0 - E_X^{total} \quad (3)$$

Optical properties

For understanding the optical properties, a parameter that has to be evaluated is the overlap integral.[1,20] and is defined as:

$$\theta = \int |r^2 dr R_e\,(r) R_h(r)|^2, \quad (4)$$

Here, $R_e(r)$ and $R_e(r)$ are the radial wavefunctions of the electron and hole, respectively, solved from the self-consistent loops. To analyze the optical transitions, we require oscillator strength, which is a unitless quantity and defined as a transition density.[21] For the exciton, oscillator strength is given as:[17]

$$f_i = \frac{2}{m_0 E} \Sigma |< u_c|p|u_v > |^2 \int |r^2 dr R_e(r) R_h(r)|^2 \quad (5)$$

Given here, u_c and u_v are defined as Bloch functions in the CB and VB, respectively, m_0 is the free electron mass and E is the excitation energy. The Kane matrix elements can be simplified as:[22]

$$|< u_{c0}|p|u_{v0} > |^2 = \frac{m_0 E_p}{4}, \quad (6)$$

where E_p is the Kane energy, 17.5 eV for CdSe, 19.6 eV for CdS.[23,24,25] From the equations (17) and (16), the electronic transition density is expressed as:

$$f = \frac{E_p}{2E} | \int r^2 dr R_e(r) R_h(r)| \quad (7)$$

And the radiative (natural) lifetime is defined as[26]:

$$\tau = \frac{6\pi\varepsilon_0 m_0 c^3}{e^2 n \beta_s E^2 f} \quad (8)$$

where, $\boldsymbol{\varepsilon_0}$ is the dielectric permittivity in vacuum, $\boldsymbol{m_0}$ is the free electron mass, ***c*** is the velocity of light, ***e*** is the electronic charge, ***f*** is the oscillator strength, ***n*** is the refractive index, ***E*** is the transition energy, and $\boldsymbol{\beta_s}$ is screening factor given as:[27]

$$\beta_s = \frac{3\varepsilon}{\varepsilon_{QD} + 2\varepsilon} \quad (9)$$

Here, ε and ε_{QD} are the optical dielectric constants of the medium and nanocrystal quantum dots, respectively. The confinement region of the exciton is determined by the overlap probability equation of the electron and hole as given by:[28]

$$Overlap\ Probability = (R_e(r) * R_h(r)) \times r^2 \quad (10)$$

Overlap percentage is another such criteria to determine the confinement region of the electron-hole overlap. Itis the relative coverage of the core/shell excitons with respect to the QDs exciton to distinguish the distribution of the excitons over the core-shell domain, which is given as[23]:

$$\text{Overlap percentage of the Type-I exciton} = \frac{\int_0^{r_{CdSe}} R_e.R_h.r^2.dr}{\int_0^{r_{CdS}} R_e.R_h.r^2.dr} \times 100 \quad (11)$$

$$\text{Overlap percentage of the Type-II exciton} = \frac{\int_{r_{CdSe}}^{r_{CdS}} R_e.R_h.r^2.dr}{\int_0^{r_{CdS}} R_e.R_h.r^2.dr} \times 100 \quad (12)$$

Here r_{CdS} and r_{CdSe} are the radius of the CdSe and CdS QDs, respectively.

Table S1. The material parameters used in the calculations

**Material**	$\boldsymbol{m_e^*}$[29]	$\boldsymbol{m_h^*}$[30]	$\boldsymbol{\kappa}$[29]	$\boldsymbol{E_g}$**(eV)**[29]	$\boldsymbol{\chi(eV)}$
**CdSe**	0.13m$_0$	0.45m$_0$	9.3	1.75	4.41
**CdS**	0.21m$_0$	0.80m$_0$	8.9	2.49	4.12

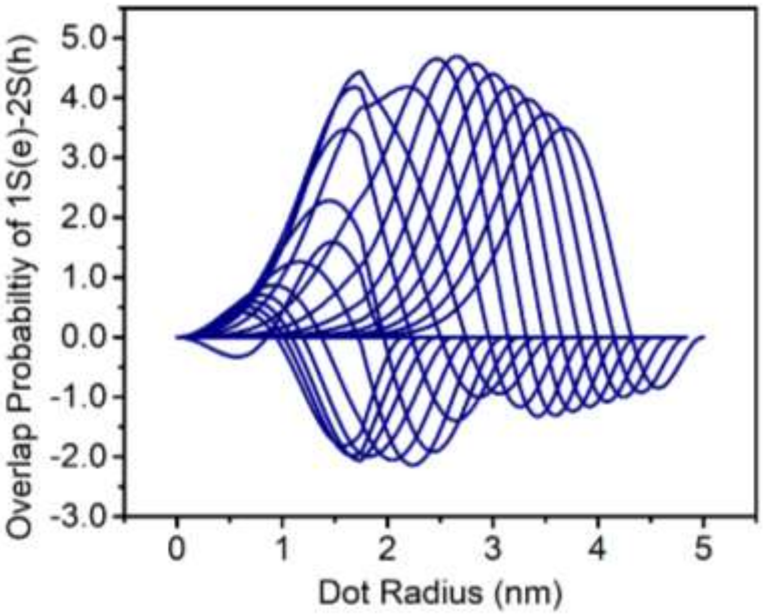

Figure S1. Overlap probability of the hole in the second sub-band of VB and electron of LUMO.

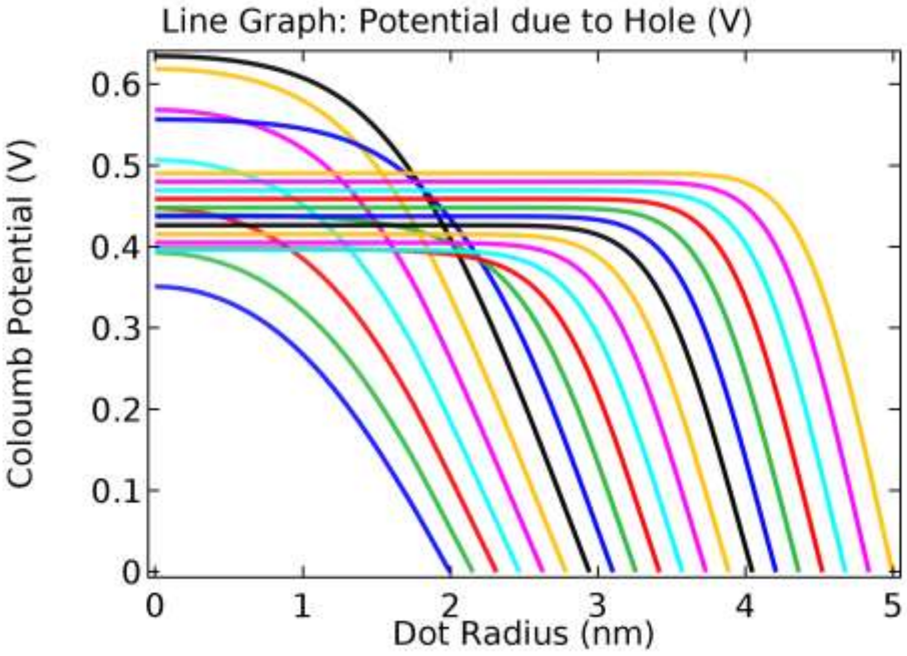

(a)

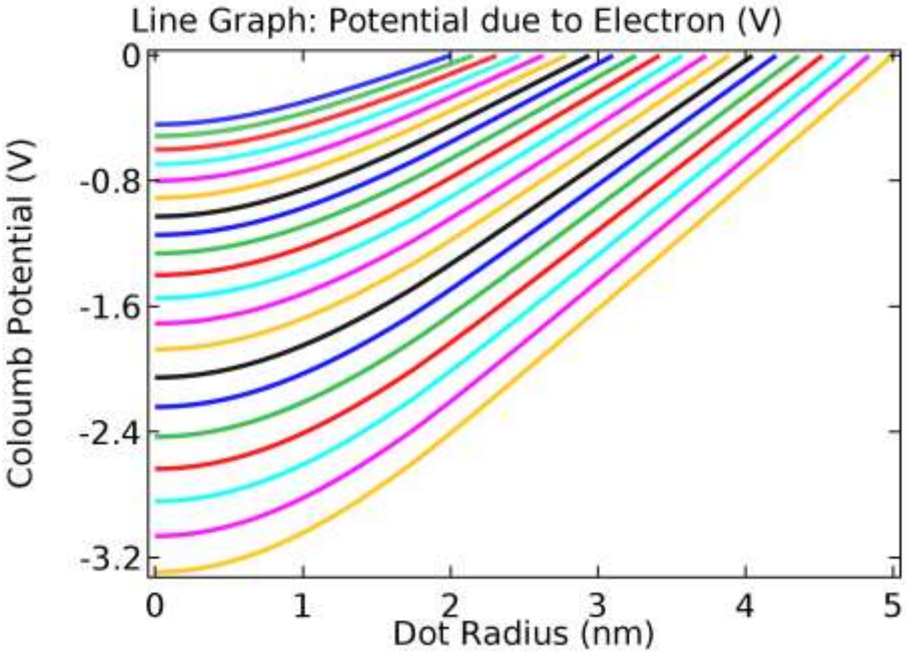

(b)

Figure S2. Variation of the Coulomb potential (a) of hole and (b) for electron with varying the shell thickness.

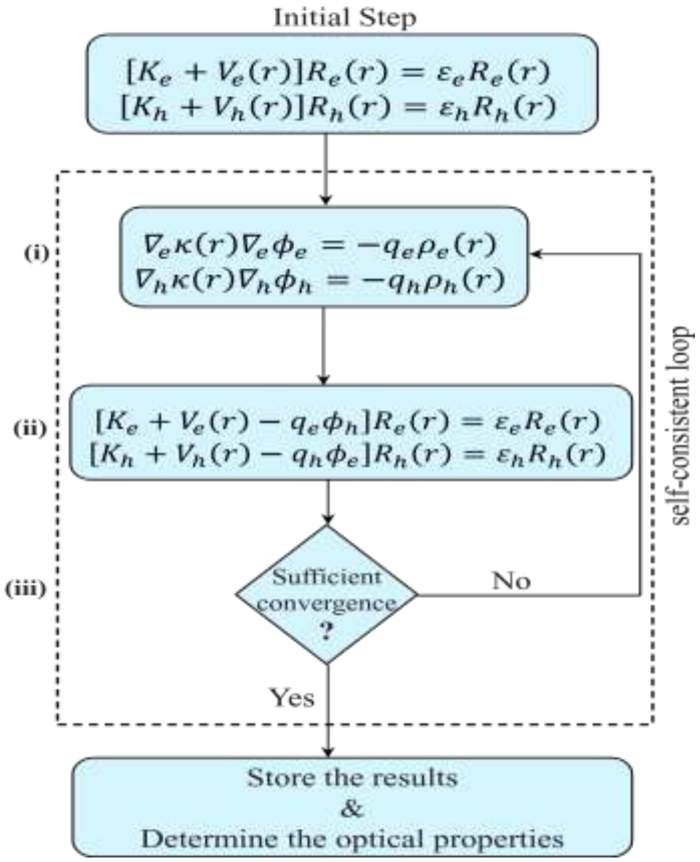

Figure S3. Algorithm for the self-consistent cycle of the mean approximation method for single exciton.

References

1. Garcia-Santamaría, F., Brovelli, S., Viswanatha, R., Hollingsworth, J. A., Htoon, H., Crooker, S. A., et al. Breakdown of volume scaling in auger recombination in CdSe/CdS heteronanocrystals: The role of the Core-Shell interface. *Nano Letters*, *11*, 687-693 (*2011*).
2. Mlinar, V., & Zunger, A. Internal electronic structure and fine structure of multiexcitons in semiconductor quantum dots. *Physical Review B, 80*, 205311 (*2009*).
3. Bester, G., Nair, S., & Zunger, A. Pseudopotential calculation of the excitonic fine structure of million-atom self-assembled $In_{1-x}Ga_xAs/GaAs$ quantum dots. *Physical Review B, 67*, 161306 (*2003*).
4. Chamarro, M., Gourdon, C., Lavallard, P., Lublinskaya, O., & Ekimov, A. I. Enhancement of electron-hole exchange interaction in CdSe nanocrystals: A quantum confinement effect. *Physical Review B, 53*, 1336-1342 (*1996*).
5. Li, J., & Wang, L. First principle study of core/shell structure quantum dots. *Applied Physics Letters, 84*, 3648-3650 (*2004*).
6. Perdew, J.P., Burke, K. and Ernzerhof, M. Generalized gradient approximation made simple. *Physical Review Letters*, *77*(18), 3865 (1996).
7. Perdew, J.P., Burke, K. and Ernzerhof, M., 1998. Perdew, burke, and ernzerhof reply. *Physical Review Letters*, *80*(4), 891 (1998).
8. Badaeva, E., et al. "Investigation of pure and Co^{2+}-doped ZnO quantum dot electronic structures using the density functional theory: choosing the right functional." *New Journal of Physics*, *10*, 055013 (2008).
9. Frisch, M.J. et al., Gaussian 09, Revision C.01, Gaussian Inc, Wallingford CT, 2010.
10. COMSOL Multiphysics reference manual, COMSOL, Inc, www.comsol.com

11. Li, J. J., Wang, Y. A., Guo, W., Keay, J. C., Mishima, T. D., Johnson, M. B., et al. Large-scale synthesis of nearly monodisperse CdSe/CdS Core/Shell nanocrystals using air-stable reagents via successive ion layer adsorption and reaction. *Journal of the American Chemical Society, 125*, 12567-12575 (*2003*).
12. MATLAB 2009, The MathWorks, Natick (2009).
13. Origin (OriginLab, Northampton, MA).
14. Åžahin, M., Nizamoglu, S., Kavruk, A. E., & Demir, H. V. Self-consistent computation of electronic and optical properties of a single exciton in a spherical quantum dot via matrix diagonalization method. *Journal of Applied Physics, 106*, 043704 (*2009*).
15. de Sousa, J. S., Freire, J. A. K., & Farias, G. A. Exciton escape in CdSe core-shell quantum dots: Implications for the development of nanocrystal solar cells. *Physical Review B, 76*, 155317 (*2007*).
16. Meulenberg, R. W., Lee, J. R., Wolcott, A., Zhang, J. Z., Terminello, L. J., & Van Buuren, T. Determination of the exciton binding energy in CdSe quantum dots. *ACS Nano, 3*, 325-330 (2009).
17. Åžahin, M., Nizamoglu, S., Kavruk, A. E., & Demir, H. V. Self-consistent computation of electronic and optical properties of a single exciton in a spherical quantum dot via matrix diagonalization method. *Journal of Applied Physics, 106*, 043704 (*2009*).
18. Tsuchiya, T. Biexcitons and charged excitons in quantum dots: A quantum Monte Carlo study. *Physica E: Low-Dimensional Systems and Nanostructures, 7,* 470-474 (*2000*).
19. Shumway, J., Franceschetti, A., & Zunger, A. Correlation versus mean-field contributions to excitons, multiexcitons, and charging energies in semiconductor quantum dots. *Physical Review B, 63,* 155316 (*2001*).
20. *Introduction,* Wiley-VCH Verlag GmbH & Co. KGaA (*2006*).

21. Ratnesh, R. K., & Mehata, M. S. Investigation of biocompatible and protein sensitive highly luminescent quantum dots/nanocrystals of CdSe, CdSe/ZnS and CdSe/CdS. *Spectrochimica Acta Part A: Molecular and Biomolecular Spectroscopy, 179,* 201-210 (*2017*).
22. Madarasz, F. L., Szmulowicz, F., Hopkins, F. K., & Dorsey, D. L. Prediction of giant χ (3) values from a calculation of excitonic nonlinear optical properties in rectangular GaAs quantum-well wires. *Physical Review B, 49*, 13528-13541 (*1994*).
23. Jaskolski, W., & Bryant, G. W. Multiband theory of quantum-dot quantum wells: Dim excitons, bright excitons, and charge separation in heteronanostructures. *Physical Review B, 57*, R4237-R4240 (*1998*).
24. Åžahin, M., Nizamoglu, S., Yerli, O., & Volkan Demir, H. Reordering orbitals of semiconductor multi-shell quantum dot-quantum well heteronanocrystals. *Journal of Applied Physics, 111*, 023713 (*2012*).
25. Dalgarno, P. A., Smith, J. M., McFarlane, J., Gerardot, B. D., Karrai, K., Badolato, A., et al. Coulomb interactions in single charged self-assembled quantum dots: Radiative lifetime and recombination energy. *Physical Review B, 77*, 245311 (*2008*).
26. Califano, M., Franceschetti, A., & Zunger, A. Lifetime and polarization of the radiative decay of excitons, biexcitons, and trions in CdSe nanocrystal quantum dots. *Physical Review B, 75*, 115401 (*2007*).
27. Martínez-Pastor, J., Bosch, J., Biswas, D., Alén, B., Valdés, J. L., Garcia, J. M., & González, L. Exciton Recombination in Self-Assembled InAs/GaAs Small Quantum Dots under an External Electric Field. *Physica Status Solidi (a), 190*, 599-603 (2002).
28. Chen, O., Zhao, J., Chauhan, V. P., Cui, J., Wong, C., Harris, D. K., & Bawendi, M. G. Compact high-quality CdSe–CdS core–shell nanocrystals with narrow emission linewidths and suppressed blinking. *Nature Materials, 12*, 445 (2013).

29. Adachi, S., *Properties of group-IV, III-V and II-VI semiconductors* John Wiley & Sons, Ltd (2005).

30. Nizamoglu, S. & Demir, H. V. Onion-like (CdSe) ZnS/CdSe/ZnS quantum-dot-quantum-well heteronanocrystals for investigation of multi-color emission. *Optics Express*, *16*, 3515 (*2008*).
